# Extubation in the operating room after elective on-pump CABG surgery: impact on patient outcome and clinical practice during the COVID-19 pandemic

**DOI:** 10.1007/s12055-025-01908-9

**Published:** 2025-03-24

**Authors:** Anna Fischbach, Julia Alexandra Simons, Steffen Bernhard Wiegand, Celiné Lang, Rüdger Kopp, Gernot Marx, Sebastian Johannes Bauer, Patrick Winnersbach, Payam Akhyari, Gereon Schälte

**Affiliations:** 1 Department of Anesthesiology, RWTH Aachen University, Aachen, 52074 Germany; 2 Department of Anesthesia and Intensive Care Medicine, Hannover Medical School, Hannover, 30625 Germany; 3 Department of Intensive Care Medicine, RWTH Aachen University, Aachen, 52074 Germany; 4 Department of Cardiothoracic Surgery, RWTH Aachen University, Aachen, 52074 Germany

**Keywords:** Early extubation, Enhanced recovery after surgery, ERAS, On-pump CABG surgery

## Abstract

**Purpose:**

Coronary artery bypass graft (CABG) surgery is the standard treatment for advanced coronary artery disease. Despite evidence supporting enhanced recovery after surgery (ERAS) programs, many hospitals continue to keep patients intubated following on-pump CABG surgery. The coronavirus disease 2019 (COVID-19) pandemic further strained intensive care unit (ICU) capacities, leading to the consideration of immediate extubation after elective surgeries like CABG surgeries. The aim of this study was to assess whether extubation in the operating room after elective on-pump CABG surgery would reduce the ICU length of stay, the ICU readmission, and the ICU mortality in a population of patients undergoing on-pump CABG surgery as opposed to the conventional approach with patients remaining intubated.

**Methods:**

This study is a retrospective single-center study, including data from the University Hospital Aachen, Germany. Clinical data from 2019 to 2022 were analyzed, focusing on patients who underwent on-pump CABG surgery. Primary endpoints studied were the duration of ICU stay, rates of ICU readmission, and ICU mortality. Secondary outcomes included the hospital length of stay, hospital mortality, and the occurrence of postoperative pneumonia.

**Results:**

Ninety-seven patients who underwent elective on-pump CABG surgery were identified. There were no variations in outcomes, including ICU and hospital stays, mortality, ICU readmission, or postoperative pneumonia between the two groups.

**Conclusion:**

Extubation in the operating room after on-pump CABG surgery did not result in significant differences in outcomes compared to patients who remained intubated.

**Graphical Abstract:**

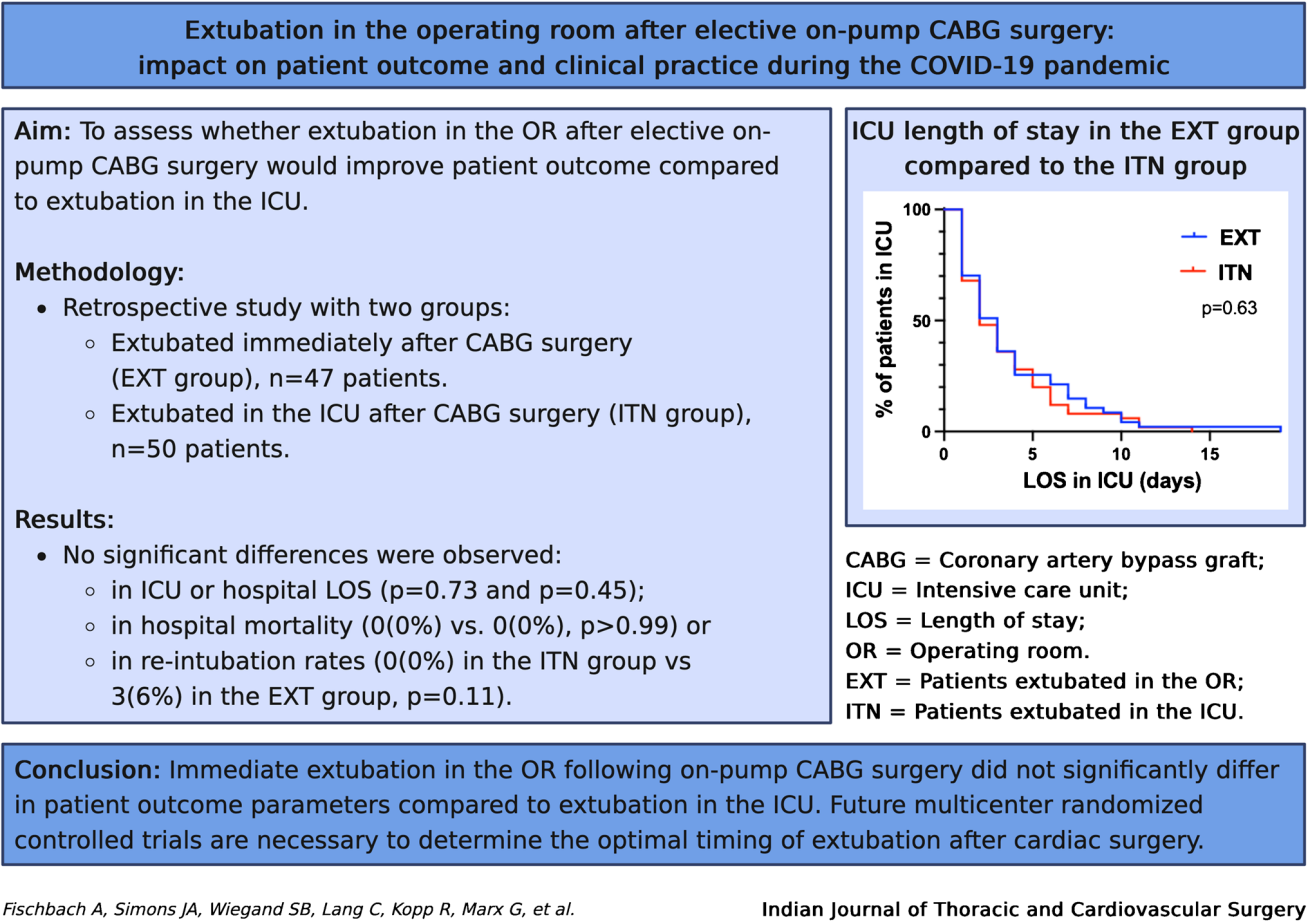

**Supplementary Information:**

The online version contains supplementary material available at 10.1007/s12055-025-01908-9.

## Introduction

Coronary artery bypass graft (CABG) surgery has emerged as the gold standard therapy for advanced coronary artery disease, predominantly performed using cardiopulmonary bypass (on-pump). The perioperative and in-hospital mortality rates associated with CABG surgery are estimated at approximately 1% for elective patients at the lowest risk, while ranging from 2 to 5% across all patient categories [1–3].

In an effort to optimize postoperative recovery and decrease intensive care unit (ICU) length of stay (LOS), a “fast-track” approach for patients undergoing cardiac surgery emerged in the early 1990s. It most notably included the optimization of anesthesia protocols as well as early extubation within anywhere between 7 and 12 h after surgery [4, 5]. Early studies on patients undergoing CABG surgery managed to demonstrate the fast-track approach to both be safe and lead to a decrease in ICU and hospital LOS [6, 7].

The subsequent introduction of enhanced recovery after surgery (ERAS) protocols in 1997 [8] marked a pivotal shift towards optimizing pre-, peri-, and postoperative patient management, aiming to accelerate postoperative recovery. This multimodal and interdisciplinary concept has been crucial in significantly reducing postoperative complications, mortality rates, and the duration of hospital stays [9, 10]. However, despite the demonstrated benefits, the adoption of ERAS protocols in cardiac surgery has been slow, with a majority of the studies highlighting their efficacy conducted within the recent 2 to 3 years [11–13]. These studies have shown that ERAS programs can decrease both the duration of hospitalization and postoperative morbidity in cardiac surgery [13, 14].

Nonetheless, the prevailing practice in many hospitals has remained to keep patients intubated rather than pursuing extubation within 6 h after on-pump CABG surgery as recommended by Engelman [15]. The onset of the coronavirus disease 2019 (COVID-19) pandemic underscored the critical need for efficient patient management strategies, such as early extubation, to optimize ICU bed availability for COVID-19 patients [16]. Early reports during the pandemic indicated that up to 20% of individuals with a COVID-19 infection developed severe illness requiring hospitalization [17–19], with a significant fraction of these patients necessitating ICU care [20, 21]. The variability in ICU admission rates in both the USA and Canada, ranging from 5 to 81% [22–24], along with the heightened demand for ICU beds, pressured hospitals during the COVID-19 pandemic to devise new strategies to alleviate healthcare system strains [25, 26]. Currently, hospitals are no longer under such strain [27].

In response, hospitals have sought to reduce the length of hospital stays for elective surgeries and implemented early extubation protocols to expedite discharge, thereby facilitating better management of surgical patients during the pandemic. This shift towards early extubation in cardiac surgery aims at shortening ICU stays and decreasing the reliance on prolonged invasive mechanical ventilation, reflecting a broader adaptation to the challenges imposed by the pandemic.

Therefore, this retrospective study was conducted to assess the feasibility and safety of extubating patients in the operating room immediately after on-pump CABG surgery, and to evaluate its impact on reducing hospital and ICU LOS, ICU readmissions, as well as ICU and hospital mortality rates.

## Materials and methods

In this retrospective study, clinical data were collected and analyzed from 2019 to 2022 for patients who underwent on-pump CABG surgery at the University Hospital Aachen, Germany. The local ethics committee approved the study protocols, with approval granted on November 13, 2023 (EK 23-331), which permitted the inclusion of data from 2019 to 2022.

### **Patients**

Eligibility for the study was based on the following inclusion criteria: (1) age of 18 years or older and (2) undergoing elective on-pump CABG surgery. Patients were excluded if they required CABG surgery due to acute conditions such as ST-elevation myocardial infarction (STEMI) or non-ST-elevation myocardial infarction (NSTEMI), underwent CABG in combination with other procedures (e.g., aortic valve replacement), receiving a type of CABG other than on-pump with cardioplegia (e.g., off-pump CABG), needed postoperative extracorporeal membrane oxygenation (ECMO), or had other cardiac pathologies like aortic valve stenosis or heart failure. Extubation in the operating room was defined as extubation directly after surgery on the operating table, instead of delaying extubation until after the transfer to the ICU.

The anesthesia regimen in cardiothoracic surgery is standardized. For induction, patients received sufentanil, propofol, and rocuronium, while sevoflurane and sufentanil were used for maintenance.

Patients were divided into two groups. The first group (EXT group) consisted of patients who were extubated immediately after on-pump CABG surgery on the operating room table, provided they were hemodynamically stable (norepinephrine <0.2 μg/kg/min, epinephrine < 0.1 μg/kg/min) and had less than 200 ml of chest tube drainage (time from chest closure until extubation). Patients also had to meet the general extubation criteria [28]. Extubation was postponed in cases of anesthesiological or surgical complications, hemodynamic instability, or pulmonary impairment. The second group (ITN group) included patients who underwent on-pump CABG surgery but were not immediately extubated post-surgery. The EXT group exclusively comprised patients from the COVID-19 pandemic years (2020–2022), while the ITN group included patients from 2019, the year before the pandemic.

**Fig. 1:**
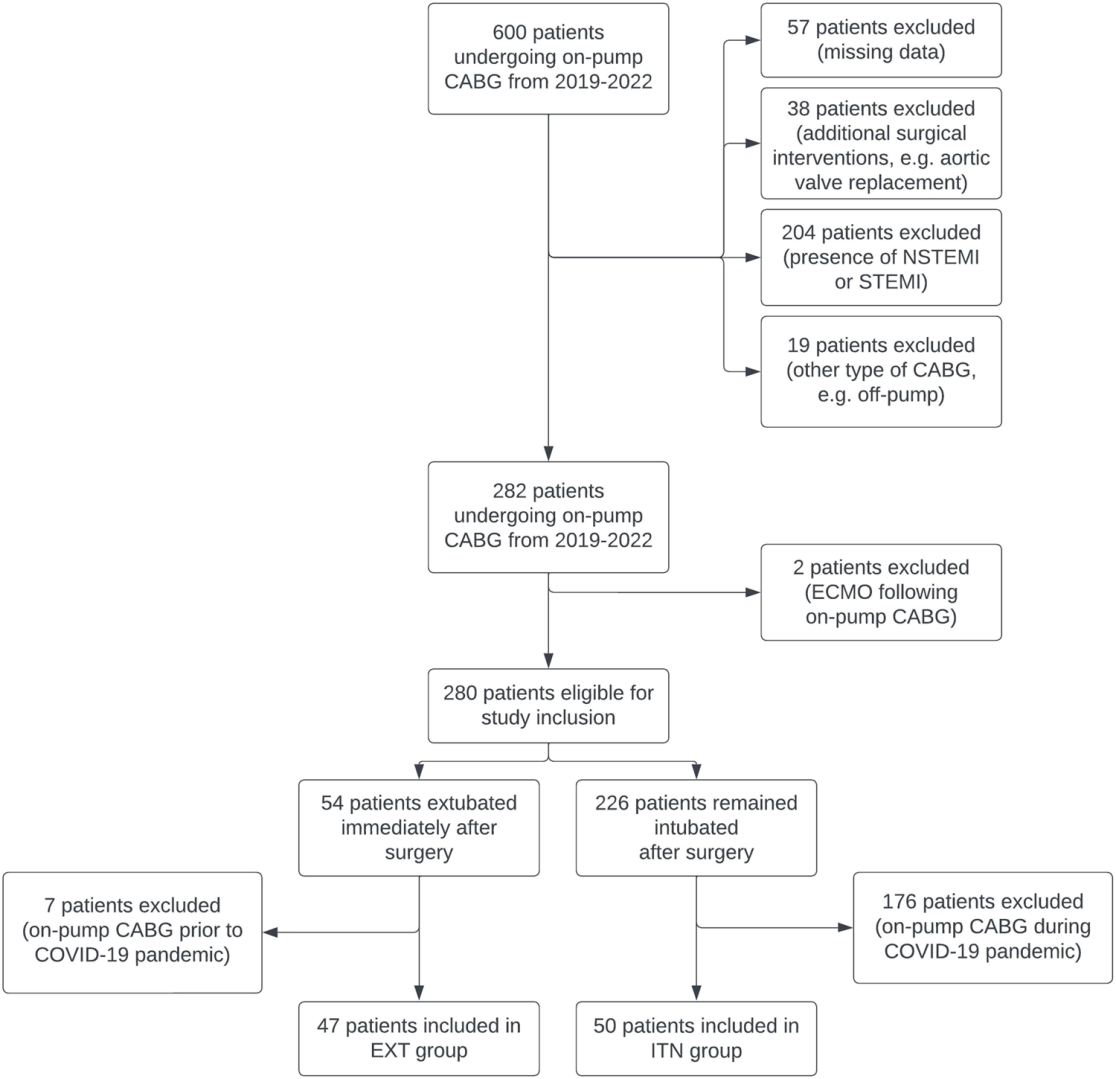
Flow chart of patient selection

Between 2019 and 2022, 600 patients underwent on-pump CABG surgery (Fig. 1). Of these, 57 patients had to be excluded due to incomplete data. An additional 38 were excluded because they received concurrent surgeries (e.g., surgical aortic valve replacement) alongside the on-pump CABG surgery. Further, 204 patients were excluded for having been diagnosed with NSTEMI or STEMI at the time of their on-pump CABG surgery. Nineteen patients were excluded due to receiving a type of CABG other than on-pump with cardioplegia (e.g., off-pump CABG). This resulted in a cohort of 282 patients, from which another 2 were excluded due to the necessity of postoperative ECMO support. Of the remaining 280 patients, 54 were extubated immediately after surgery, while 226 remained intubated. In this study, the ITN group includes only patients from *before* the COVID-19 pandemic, while the EXT group includes only patients from *during* the pandemic. Consequently, additional patients had to be excluded based on these criteria: Therefore, 176 patients from the ITN group who underwent on-pump CABG surgery during the COVID-19 pandemic, rather than before, were excluded, resulting in a final count of 50 patients for this group. Similarly, 7 patients from the EXT group who had their surgeries before and not during the pandemic, were also excluded, leaving a total of 47 patients in that group.

### **Study endpoints**

The co-primary outcomes for comparing the two groups were ICU LOS, ICU readmission, and ICU mortality. Secondary outcomes included hospital LOS, hospital mortality, rate of reintubation, and the incidence of postoperative pneumonia.

### **Statistical analysis**

Statistical analyses and graph design were conducted using GraphPad Prism software (Version 9.3.1, GraphPad Software, San Diego, CA, USA). A *p* value of less than 0.05 was considered statistically significant. Data were presented as median (first quartile–third quartile) or number (percentage). The Shapiro-Wilk test was used to assess normal distribution. For normally distributed data, an unpaired *t*-test was applied, while the Mann-Whitney test was used for data that were not normally distributed. Additional statistical data can be found in Supplementary Table 1. Outcome analysis was performed by using log-rank test with hazard ratios calculated with the Mantel-Haenszel method. Kaplan-Meier curves were used to illustrate the analysis.

## Results

### Baseline clinical characteristics of patients undergoing elective on-pump CABG surgery

Ninety-seven patients who met the inclusion criteria were included in this study. They were predominantly male (80%) with a median age of 66 years (61–71) (Table 1; Supplementary Table 1). The clinical characteristics of patients who were extubated in the operating room after surgery (EXT group) were comparable with those who remained intubated (ITN group), except for the duration of invasive mandatory ventilation (i-MV), which was longer in patients in the ITN group (8 h (6–11.6 h) vs. 0 h (0–0)), *p* < 0.01). The time spent during the surgery was not included in the total time calculated for invasive mechanical ventilation (i-MV). Notably, 6 out of 50 patients in the ITN group were extubated within 6 h after surgery.

**Table 1:** Baseline clinical characteristics of patients undergoing on-pump CABG surgery

Characteristic	All patients *n* = 97	Intubated after surgery (ITN) *n* = 50	Extubated after surgery (EXT) *n* = 47	*p* value
Age, y	66 (61–71)	67 (63–70)	65 (55–72)	0.28
Male sex	78 (80%)	40 (80%)	38 (81%)	0.92
BMI, kg/m^2^	27.1 (24.6–31.5)	26.9 (24.3–31.6)	27.4 (24.7–31.3)	0.85
SVCAD	2 (2%)	1 (2%)	1 (2%)	>0.99
DVCAD	28 (29%)	12 (24%)	16 (34%)	0.37
TVCAD	66 (68%)	36 (72%)	30 (64%)	0.51
1 bypass	2 (2%)	1 (2%)	1 (2%)	>0.99
2 bypasses	28 (29%)	13 (26%)	15 (32%)	0.65
3 bypasses	52 (54%)	26 (52%)	26 (55%)	0.84
> 3 bypasses	15 (15%)	10 (20%)	5 (11%)	0.27
Diabetes mellitus	29 (30%)	11 (22%)	18 (38%)	0.12
Pulmonary disease	10 (10%)	4 (8%)	6 (13%)	0.52
Pathological pulmonary function test^a^	9 (9%)	4 (8%)	5 (11%)	0.74
Preoperative left ventricular ejection fraction (%)	60 (53–60)	60 (53–60)	59 (53–60)	0.29
Postoperative left ventricular ejection fraction (%)^b^	55 (49–60)	55 (49–55)	56 (50–60)	0.07
Positive COVID-19 test^c^	0 (0%)	Not available	0 (0%)	n.a.
Length of surgery, h	5 (4.5–6)	5 (4.5–6)	5 (4.5–6)	0.45
Bypass time, min	97 (78–123)	106 (83–128)	97 (76–114)	0.34
Length of aortic crossclamping, min^d^	59 (50–76)	62 (49–77)	56 (50–76)	0.51
Perioperative complications^e^	11 (11%)	6 (12%)	5 (11%)	>0.99
EURO II score, %^f^	1.17 (0.8–2.3)	1.37 (0.9–2.4)	0.99 (0.7–2.1)	0.30
SOFA score	1 (0–1)	1 (0–1)	1 (0–2)	0.52
Duration i-MV	4 (0–8.5)	8.0 (6.0–11.6)	0 (0–0)^g^	< 0.01*

Results are expressed as median (first quartile–third quartile) or no. (%). The unpaired *t*-test was applied to variables with a normal distribution, specifically bypass time, while the Mann-Whitney test was used for all other variables. For variables where an unpaired *t*-test was applied, mean ± SD (standard deviation) was also calculated (Supplementary Table 1). Statistical significance was defined as *p* > 0.05

*BMI*, body mass index; *SVCAD/DVCAD/TVCAD*, single/double/three vessel coronary artery disease; *Euro II SCORE*, European System for Cardiac Operative Risk Evaluation; *SOFA score*, Sequential Organ Failure Assessment score; *i-MV*, invasive mandatory ventilation, also including ventilation times after reintubation; *n.a.*, not applicable

^a^Pathological if severe obstructive or restrictive pulmonary disease was detected

^b^Due to incomplete documentation, the postoperative left ventricular ejection fraction could not be determined for 4 out of 50 patients in the ITN group, and for 14 out of 47 patients in the EXT group

^c^Since patients in the ITN group underwent surgery in 2019, prior to the availability of COVID-19 testing, data on their COVID-19 status was not available

^d^Due to incomplete documentation, the length of aortic crossclamping could not be included for 3 out of 50 patients in the ITN group, and for 3 out of 47 patients in the EXT group

^e^Perioperative complications include ventricular fibrillation, atrial fibrillation, atrioventricular block, and in one case damage to the pulmonary artery

^f^Due to incomplete documentation, the EURO II score could be calculated for 47 out of 50 patients in the ITN group, and for 44 out of 47 patients in the EXT group

^g^The mean time between the end of surgery and extubation in the operating room was 18.62 ± 17.28 min

### Clinical characteristics 24 h after on-pump CABG surgery

**Fig. 2:**
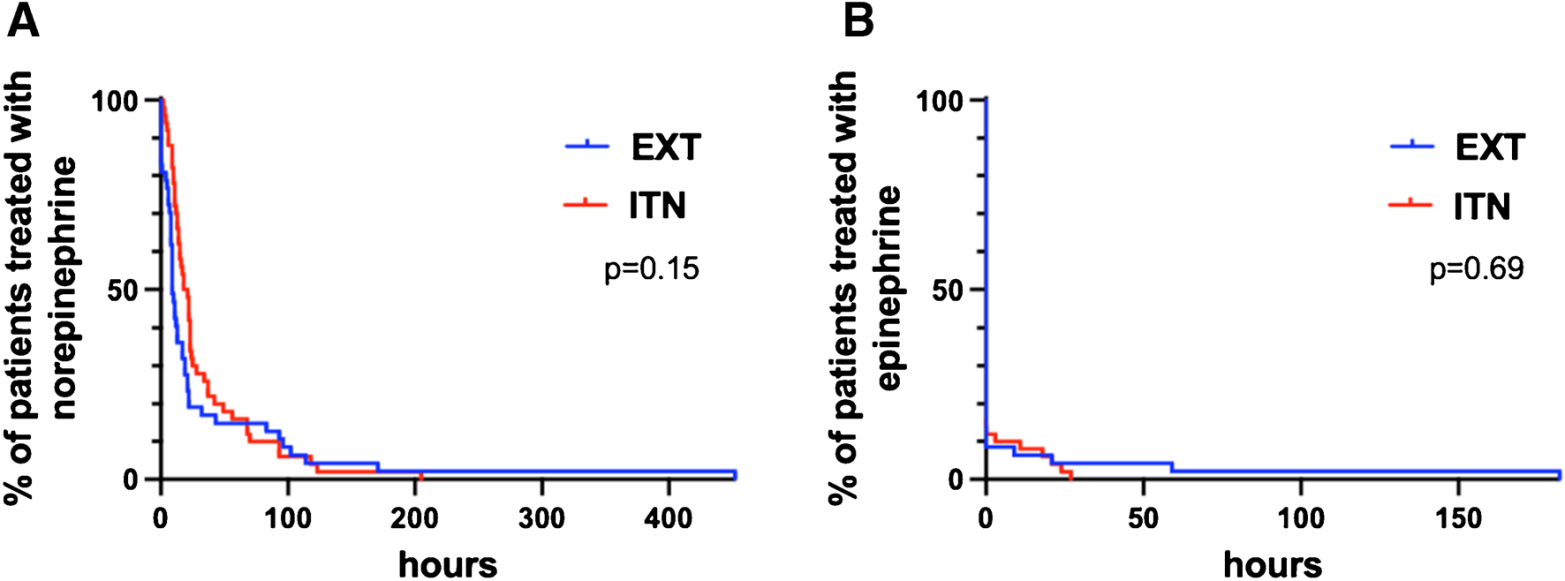
Percentage (%) of patients in the EXT and ITN group with the duration of norepinephrine administration (**A**) and duration of epinephrine administration (**B**). EXT, group of patients extubated in the operating room after surgery; ITN, group of patients who remained intubated post-surgery. There was no significant difference in the discontinuation rates of norepinephrine or epinephrine between the EXT group and the ITN group

At 24 h after on-pump CABG surgery, the EXT group required a significantly lower concentration of norepinephrine (0.000 μg/kg/min (0.000–0.000)) compared to the ITN group (0.000 μg/kg/min (0.000–0.012)), with a significant difference (*p* < 0.01) as shown in Table 2. The concentration of epinephrine needed at 24 h post-surgery was similar between both groups. The analysis also indicated that the duration for which norepinephrine or epinephrine was administered did not significantly differ between intubated and extubated patients as depicted in Fig. 2A and B (norepinephrine: *p* = 0.15; HR (hazard ratio), 1.4; CI, 0.9–2.1; epinephrine: *p* = 0.69; HR, 0.8; CI, 0.3–2.1). Furthermore, there were no significant differences in mean arterial pressure (MAP) and pulmonary oxygenation (P_a_O_2_/F_i_O_2_ ratio) between the groups at 24 h after surgery. The incidence of postoperative complications, such as delirium and atrial fibrillation, as well as the rate of reintubation, showed no significant difference between the ITN and EXT groups.

**Table 2:** Clinical characteristics 24 h after on-pump CABG surgery and incidence of postoperative complications

Characteristic	All patients *n* = 97	Intubated after surgery (ITN) *n* = 50	Extubated after surgery (EXT) *n* = 47	*p* value
24 h after surgery				
P_a_O_2_/F_i_O_2_, mmHg	273 (214–328)	279 (224–315)	267 (210–330)	0.56
p_a_CO_2_, mmHg	40 (38–43)	40 (39–43)	40 (37–42)	0.29
MAP	79 (71–86)	79 (71–87)	79 (70–85)	0.38
HR	89 (81–91)	89 (84–90)	89 (74–93)	0.75
Use of norepinephrine ug/kg/min	0.000 (0.000–0.003)	0.000 (0.000–0.012)	0 (0.000–0.000)	< 0.01*
Use of epinephrine ug/kg/min	0 (0.000–0.000)	0 (0.000–0.000)	0 (0.000–0.000)	0.24
Postoperative complications				
Perioperative complications	11 (11%)	6 (12%)	5 (11%)	>0.99
Delirium	14 (14%)	8 (16%)	6 (13%)	0.78
Atrial fibrillation	20 (21%)	10 (20%)	10 (21%)	>0.99
Pneumothorax	6 (6%)	2 (4%)	4 (9%)	0.43
Bleeding	8 (8%)	2 (4%)	6 (13%)	0.15
Pleural effusion	21 (22%)	13 (26%)	8 (17%)	0.33
Other brady- or tachyarrhythmia	9 (9%)	4 (8%)	5 (11%)	0.74

Results are expressed as median (first quartile–third quartile) or no. (%). The Mann-Whitney test was applied to all non-normally distributed variables. However, the unpaired *t*-test was used for the Horowitz index (P_a_O_2_/F_i_O_2_) and MAP at 24 h after surgery, as these variables were normally distributed. For variables where an unpaired *t*-test was applied, mean ± SD was also calculated (Supplementary Table 1). Statistical significance was defined as *p* > 0.05

*p* > 0.05 *P*_*a*_*O*_*2*_*/F*_*i*_*O*_*2*_, Horowitz index; *MAP*, mean arterial pressure; *HR*, heart rate

### Primary and secondary outcomes

**Fig. 3:**
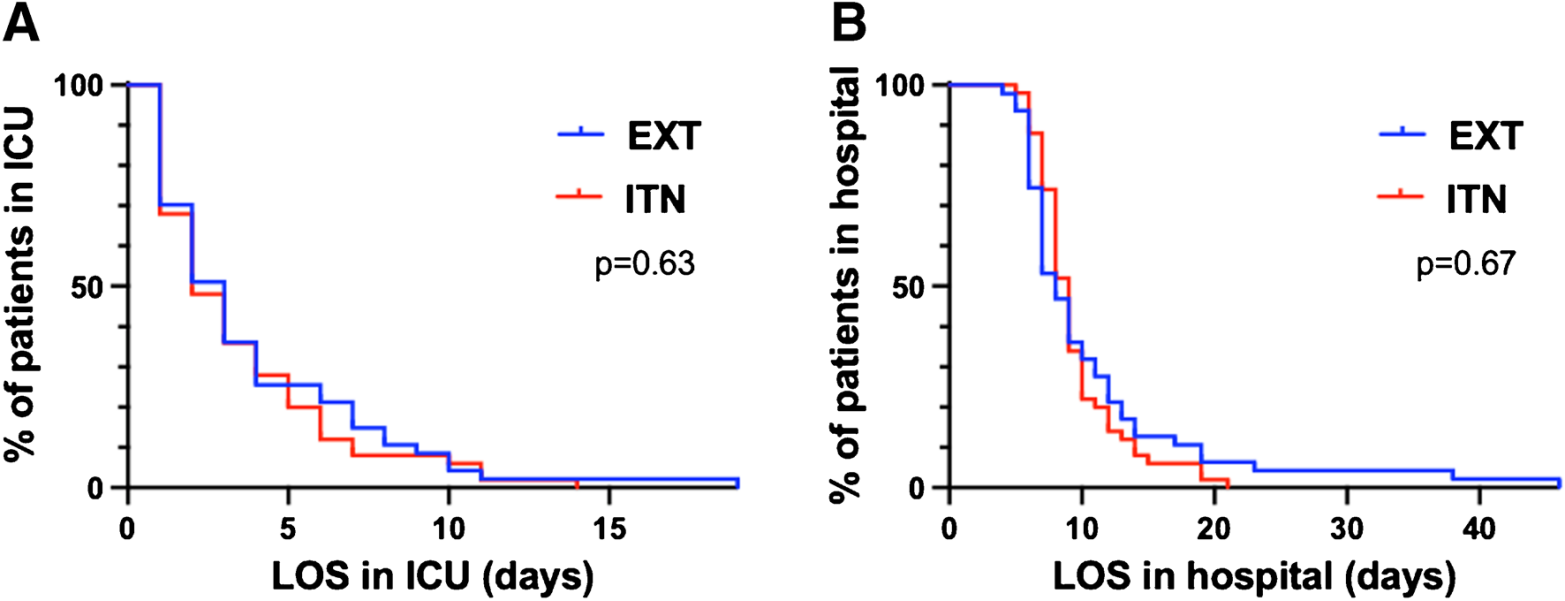
LOS in hospital and in the ICU of patients in the EXT and ITN group. **A** LOS in the hospital of patients in the EXT and ITN group. **B** LOS in the ICU of patients in the EXT and ITN group. LOS, length of stay; EXT, group of patients extubated in the operating room after surgery; ITN, group of patients who remained intubated post-surgery. Cox regression analysis showed that there was no significant association between the ITN and EXT groups regarding the rate of discharge from the ICU or hospital

No significant differences were observed in ICU or hospital LOS, mortality, reintubation rates, ICU readmission rates, or the incidence of postoperative pneumonia between the groups, as shown in Table 3. Cox regression analysis revealed no significant association between the groups (intubated vs. extubated) regarding LOS in ICU (*p* = 0.63; HR, 0.9; CI (confidence interval), 0.6–1.4) or LOS in hospital (*p* = 0.67; HR, 0.9; CI, 0.6–1.4), as shown in Fig. 3A and B.

**Table 3:** Primary and secondary outcomes

	All patients *n* = 97	Intubated after surgery (ITN) *n* = 50	Extubated after surgery (EXT) *n* = 47	*p* value
Primary outcomes				
ICU LOS, median (interquartile range)	2 (1–5)	2 (1–5)	3 (1–6)	0.73
ICU readmissions, *n* (%)	3 (3%)	2 (4%)	1 (2%)	>0.99
ICU mortality, *n* (%)	0 (0%)	0 (0%)	0 (0%)	>0.99
Secondary outcomes				
Hospital LOS, median (interquartile range)	8 (7–11)	9 (7–10)	8 (6–12)	0.45
Hospital mortality, *n* (%)	0 (0%)	0 (0%)	0 (0%)	>0.99
Reintubation, *n* (%)^a^	3 (3%)	0 (0%)	3 (6%)	0.11
Postoperative pneumonia, *n* (%)	3 (3%)	1 (2%)	2 (4%)	0.61

Results are expressed as median (first quartile–third quartile) or no. (%). *LOS*, length of stay. The Mann-Whitney test was applied to all variables. Statistical significance was defined as *p* > 0.05

^a^Reasons for reintubation: one patient had to be reintubated due to postoperative hypercapnia, one patient for a blocked left main bronchus accompanied by pneumothorax and extensive atelectasis, and a third for hypoxia of unclear etiology. None of these patients had a COVID-19 infection

## Discussion

This study evaluates the outcomes of patients undergoing on-pump CABG surgery, focusing on the effects of immediate extubation in the operating room versus conventional practices of remaining intubated post-surgery. We retrospectively collected and analyzed data from patients treated during the 2019–2022 period, including the COVID-19 pandemic years, to investigate the impact of extubation protocols on post-surgical recovery and the use of healthcare resources.

Patients who were extubated in the operating room immediately after on-pump CABG surgery (EXT group) demonstrated a shorter duration of invasive mandatory ventilation and significantly lower norepinephrine levels 24 h post-surgery compared to those who remained intubated (ITN group). There were no significant differences in ICU and hospital LOS, mortality, reintubation rates, ICU readmission rates, or the incidence of postoperative pneumonia between the two groups.

In the ITN group, 6 out of 50 patients were not extubated directly in the operating room but were extubated within 6 h postoperatively, as recommended by current ERACS (enhanced recovery after cardiac surgery) guidelines [15]. However, 44 out of 50 patients remained intubated for longer than 6 h, which does not meet these guidelines. It is important to highlight that the primary focus of this study was to explore the potential benefits of immediate on-table extubation, rather than extubation within the 6-h window recommended for post-cardiac surgery.

In addition, during the COVID-19 pandemic, ICU capacity constraints led to the implementation of direct extubation in the operating room after elective cardiac surgery to accommodate the increased need for ICU beds for COVID-19 patients. Before the pandemic, direct operating room extubation was not practiced; instead, patients were typically extubated several hours later in the ICU. Consequently, there were only very few patients meeting the criteria for the EXT group in the pre-pandemic era. Similarly, selecting intubated patients from the pandemic period would have introduced bias, as those who remained intubated were often patients with specific medical conditions requiring prolonged ventilation.

Further, none of the patients in the EXT group had a confirmed COVID-19 infection before, during, or after the CABG surgery, as all tested negative for severe acute respiratory distress syndrome coronavirus 2 (SARS-CoV-2). Since the patients in the ITN group were operated on in 2019, before COVID-19 testing was available, no data on their COVID-19 status is available.

Evidence suggests that on-table extubation [29, 30] or early extubation within 6 h post-cardiac surgery can decrease the rate of postoperative complications compared to prolonged intubation [31]. While one clinical trial found no difference in the incidence of reintubation, stroke, and renal failure between early and late extubation [31], other studies have indicated potential benefits, including reduced risks of postoperative atrial arrhythmia when extubated within 1 h after ICU arrival [32], and lower risks of pneumonia and bacteremia [33]. Meta-analyses have further supported that early extubation, within 8 h, is not associated with increased morbidity or mortality [34, 35]. Early extubation benefits include enhanced pulmonary secretion clearance, earlier mobilization, and improved cardiac performance, while prolonged mechanical ventilation is associated with higher rates of bacterial colonization and hospital-acquired pneumonia, as well as an increased risk of postoperative delirium, which has been linked to increased mortality and prolonged hospital LOS [36, 37]. Another study demonstrated that several factors are associated with early extubation, such as younger age and lower body mass index (BMI) [38]. Finally, e Silva and Badhwar et al. explored the financial implications of extubation practices, revealing that extubation in the operating room can significantly reduce hospital costs by minimizing the need for prolonged ICU monitoring and associated staffing costs, in addition to reducing ventilator-associated complications [39, 40].

At our hospital, patients were not extubated immediately after cardiac surgery per protocol but were transferred to the ICU while still intubated. The pandemic necessitated a shift in practice, with anesthesiologists opting to extubate patients to reduce ICU stays and make room for COVID-19 patients requiring intensive care. This study included 47 patients in the EXT group, with a similar number from 2019 selected for the ITN group to ensure comparable baseline characteristics, revealing no significant differences between the groups in this regard.

Notably, patients in the ITN group required significantly higher doses of norepinephrine compared to those in the EXT group. This difference is likely attributable to the need for sedation in intubated patients, as sedative agents can significantly impact hemodynamic stability. Consequently, additional vasopressors are often necessary to maintain adequate MAP.

Prior to the COVID-19 pandemic, routine on-table extubation was not a common practice from the perspective of cardiac surgeons and was not considered a standard approach (“lex chirurgica”). As a result, it was not a topic of serious discussion within our institution. However, the pandemic brought increased restrictions and a persistent shortage of ICU beds, which led to a significant shift in mindset. This situation prompted the initiation of an early extubation program, during which cardiac surgeons began to recognize the advantages of early extubation.

As the pandemic subsided, the benefits of early extubation became widely accepted among cardiac surgeons, who advocated for it to become the new standard of care. The primary adjustment during this period was the implementation of on-tabula extubation. Additionally, adjustments were made in anesthetic management, such as switching from intraoperative sufentanil to remifentanil and introducing intraoperative dexmedetomidine to reduce the use of sevoflurane and potentially lower the risk of postoperative cognitive deficits.

These changes, initially driven by the pandemic, have demonstrated benefits that extend beyond the immediate crisis and have now been integrated into current practice.

### Limitations

It must be acknowledged that this study is subject to limitations. First, the retrospective design introduces potential biases, including selection bias and the retrospective collection of data, which may affect the reliability of the outcomes observed. The method of data collection and analysis relies on the accuracy and completeness of medical records, which might not capture all nuances of patient care or subtle clinical indicators that could influence outcomes.

Secondly, the study cohort was drawn from a single institution, which may limit the generalizability of the findings to other settings. Practices, patient demographics, and healthcare resources can vary significantly across different institutions and geographical locations, potentially influencing the applicability of the results to broader populations. The unique protocols and patient management strategies of the participating hospital may not be representative of those employed in other hospitals or cardiac surgery centers.

Another critical limitation is the selection of patients for the ITN group from the year 2019, exclusively before the COVID-19 pandemic, as opposed to the EXT group where patients were selected from the pandemic period (2020–2022). This distinction is pivotal as patients who remained intubated during the pandemic likely did so out of necessity, such as deteriorating gas exchange, which could inadvertently introduce a higher risk of selection bias into the study. Despite these challenges, a thorough comparison of baseline characteristics—including age, gender, BMI, comorbidities like pulmonary disease or diabetes mellitus, Sequential Organ Failure Assessment score (SOFA score), and European System for Cardiac Operative Risk Evaluation (Euro SCORE II)—revealed no significant differences between the groups, thereby facilitating a robust comparative analysis.

The relatively small sample size of this study of 97 patients limits the statistical power to detect small but clinically significant differences between groups. This limitation is particularly relevant for outcomes that occur infrequently, where a larger sample size might have provided the necessary power to observe significant differences. The inclusion criteria, designed to select patients during the COVID-19 pandemic for the EXT group, further constrained the ability to increase the sample size of this study, as it was limited by the number of eligible patients treated during this specific period.

Additionally, our analysis did not include a power calculation, primarily because we included all eligible patients for the EXT group from the pandemic period. Given that our study was conducted at a single center, we were unable to expand our patient sample size beyond those who met our inclusion criteria during the specified timeframe.

Lastly, the focus on short-term outcomes, such as ICU and hospital stay, without assessing longer-term effects or patient-reported outcomes, limits the scope conclusions of the study. Long-term recovery, quality of life post-surgery, and patient satisfaction are critical dimensions of surgical success that were not addressed in this analysis.

Future research should aim to address these limitations by employing prospective, multicenter study designs with larger, more diverse patient populations to strengthen the reliability and generalizability of the findings.

## Conclusion

In conclusion, although immediate extubation in the operating room following on-pump CABG surgery did not show significant differences in ICU or hospital LOS, mortality, or ICU readmission rates compared to conventional intubation practices, it may still offer potential benefits, such as reduced norepinephrine use. Future multicenter randomized controlled trials are necessary to determine the optimal timing of extubation after cardiac surgery to enhance patient outcomes and healthcare efficiency.

## Supplementary Information

Below is the link to the electronic supplementary material.

Supplementary file1 (DOCX 405 KB)

Supplementary file2 (MP4 9.12 MB)

## Data Availability

The datasets generated and/or analyzed during the current study are available from the corresponding author upon reasonable request.

## References

[1] Hannan EL, Racz MJ, Walford G, Jones RH, Ryan TJ, Bennett E, et al. Long-term outcomes of coronary-artery bypass grafting versus stent implantation. N Engl J Med. 2005;352:2174–83. 15917382 10.1056/NEJMoa040316

[2] Birkmeyer JD, Siewers AE, Finlayson EVA, Stukel TA, Lucas FL, Batista I, et al. Hospital volume and surgical mortality in the United States. N Engl J Med. 2002;346:1128–37. 11948273 10.1056/NEJMsa012337

[3] Peterson ED, Coombs LP, DeLong ER, Haan CK, Ferguson TB. Procedural volume as a marker of quality for CABG surgery. JAMA. 2004;291:195–201. 14722145 10.1001/jama.291.2.195

[4] Engelman RM, Rousou JA, Flack JE, Deaton DW, Humphrey CB, Ellison LH, et al. Fast-track recovery of the coronary bypass patient. Ann Thorac Surg. 1994;58:1742–6. 7979747 10.1016/0003-4975(94)91674-8

[5] Arom KV, Emery RW, Petersen RJ, Schwartz M. Cost-effectiveness and predictors of early extubation. Ann Thorac Surg. 1995;60:127–32. 7598574

[6] Cheng DCH, Karski J, Peniston C, Asokumar B, Raveendran G, Carroll J, et al. Morbidity outcome in early versus conventional tracheal extubation after coronary artery bypass grafting: a prospective randomized controlled trial. J Thorac Cardiovasc Surg. 1996;112:755–64. 8800165 10.1016/S0022-5223(96)70062-4

[7] Engelman RM. Mechanisms to reduce hospital stays. Ann Thorac Surg. 1996;61:33–4. 8572829 10.1016/0003-4975(95)01081-5

[8] Kehlet H. Multimodal approach to control postoperative pathophysiology and rehabilitation. Br J Anaesth. 1997;78:606–17. 9175983 10.1093/bja/78.5.606

[9] Spanjersberg WR, Reurings J, Keus F, van Laarhoven CJ. Fast track surgery versus conventional recovery strategies for colorectal surgery. Cochrane Database Syst Rev. 2011;2:CD007635. 10.1002/14651858.CD007635.pub2 PMC13061361 21328298

[10] Bond-Smith G, Belgaumkar AP, Davidson BR, Gurusamy KS. Enhanced recovery protocols for major upper gastrointestinal, liver and pancreatic surgery. Cochrane Database Syst Rev. 2016;2:CD011382. 26829903 10.1002/14651858.CD011382.pub2 PMC8765738

[11] Berretta P, De Angelis V, Alfonsi J, Pierri MD, Malvindi PG, Zahedi HM, et al. Enhanced recovery after minimally invasive heart valve surgery: early and midterm outcomes. Int J Cardiol. 2023;370:98–104. 36375597 10.1016/j.ijcard.2022.11.016

[12] Mondal S, Bergbower EAS, Cheung E, Grewal AS, Ghoreishi M, Hollander KN, et al. Role of cardiac anesthesiologists in intraoperative enhanced recovery after cardiac surgery (ERACS) protocol: a retrospective single-center study analyzing preliminary results of a yearlong ERACS protocol implementation. J Cardiothorac Vasc Anesth. 2023;37:2450–60. 36517338 10.1053/j.jvca.2022.11.007

[13] Grant MC, Isada T, Ruzankin P, Whitman G, Lawton JS, Dodd-o J, et al. Results from an enhanced recovery program for cardiac surgery. J Thorac Cardiovasc Surg. 2020;159:1393–1402.e7. 10.1016/j.jtcvs.2019.05.035 31279510

[14] Markham T, Wegner R, Hernandez N, Lee JW, Choi W, Eltzschig HK, et al. Assessment of a multimodal analgesia protocol to allow the implementation of enhanced recovery after cardiac surgery: retrospective analysis of patient outcomes. J Clin Anesth. 2019;54:76–80. 30412813 10.1016/j.jclinane.2018.10.035

[15] Engelman DT, Ben Ali W, Williams JB, Perrault LP, Reddy VS, Arora RC, et al. Guidelines for perioperative care in cardiac surgery: enhanced recovery after surgery society recommendations. JAMA Surg. 2019;154:755–66. 31054241 10.1001/jamasurg.2019.1153

[16] George I, Salna M, Kobsa S, Deroo S, Kriegel J, Blitzer D, et al. The rapid transformation of cardiac surgery practice in the coronavirus disease 2019 (COVID-19) pandemic: insights and clinical strategies from a center at the epicenter. Ann Thorac Surg. 2020;110:1108–18. 32591132 10.1016/j.athoracsur.2020.04.012 PMC7309733

[17] Huang C, Wang Y, Li X, Ren L, Zhao J, Hu Y, et al. Clinical features of patients infected with 2019 novel coronavirus in Wuhan, China. Lancet. 2020;395:497–506. 31986264 10.1016/S0140-6736(20)30183-5 PMC7159299

[18] Mahase E. Covid-19: most patients require mechanical ventilation in first 24 hours of critical care. BMJ. 2020;368:m1201. 32209544 10.1136/bmj.m1201

[19] Wang D, Hu B, Hu C, Zhu F, Liu X, Zhang J, et al. Clinical characteristics of 138 hospitalized patients with 2019 novel coronavirus-infected pneumonia in Wuhan, China. JAMA. 2020;323:1061–9. 32031570 10.1001/jama.2020.1585 PMC7042881

[20] Yang X, Yu Y, Xu J, Shu H, Xia J, Liu H, et al. Clinical course and outcomes of critically ill patients with SARS-CoV-2 pneumonia in Wuhan, China: a single-centered, retrospective, observational study. Lancet Respir Med. 2020;8:475–81. 32105632 10.1016/S2213-2600(20)30079-5 PMC7102538

[21] Wu Z, McGoogan JM. Characteristics of and important lessons from the coronavirus disease 2019 (COVID-19) outbreak in China: summary of a report of 72 314 cases from the Chinese Center for Disease Control and Prevention. JAMA. 2020;323:1239–42. 32091533 10.1001/jama.2020.2648

[22] Richardson S, Hirsch JS, Narasimhan M, Crawford JM, McGinn T, Davidson KW, et al. Presenting characteristics, comorbidities, and outcomes among 5700 patients hospitalized with COVID-19 in the New York City area. JAMA. 2020;323:2052–9. 32320003 10.1001/jama.2020.6775 PMC7177629

[23] Arentz M, Yim E, Klaff L, Lokhandwala S, Riedo FX, Chong M, et al. Characteristics and outcomes of 21 critically ill patients with COVID-19 in Washington State. JAMA. 2020;323:1612–4. 32191259 10.1001/jama.2020.4326 PMC7082763

[24] Murthy S, Archambault PM, Atique A, Carrier FM, Cheng MP, Codan C, et al. Characteristics and outcomes of patients with COVID-19 admitted to hospital and intensive care in the first phase of the pandemic in Canada: a national cohort study. CMAJ Open. 2021;9:E181-8. 33688026 10.9778/cmajo.20200250 PMC8034299

[25] French G, Hulse M, Nguyen D, Sobotka K, Webster K, Corman J, et al. Impact of hospital strain on excess deaths during the COVID-19 pandemic-United States, July 2020-july 2021. Am J Transplant. 2022;22:654–7. 35113490 10.1111/ajt.16645 PMC9811904

[26] Davis B, Bankhead-Kendall BK, Dumas RP. A review of COVID-19’s impact on modern medical systems from a health organization management perspective. Health Technol (Berl). 2022;12:815–24. 35371904 10.1007/s12553-022-00660-z PMC8956330

[27] Cucinotta D, Vanelli M. WHO declares COVID-19 a pandemic. Acta Bio Medica Atenei Parmensis. 2020;91:157–60. 32191675 10.23750/abm.v91i1.9397 PMC7569573

[28] Miller KA, Harkin CP, Bailey PL. Postoperative tracheal extubation. Anesth Analg. 1995;80:149–72. 7802273 10.1097/00000539-199501000-00025

[29] Kintrup S, Malec E, Kiski D, Schmidt C, Brünen A, Kleinerüschkamp F, et al. Extubation in the operating room after Fontan procedure: does it make a difference? Pediatr Cardiol. 2019;40:468–76. 30238137 10.1007/s00246-018-1986-5

[30] Jaquet O, Gos L, Amabili P, Donneau AF, Mendes MA, Bonhomme V, et al. On-table extubation after minimally invasive cardiac surgery: a retrospective observational pilot study. J Cardiothorac Vasc Anesth. 2023;37:2244–51. 37612202 10.1053/j.jvca.2023.07.037

[31] Richey M, Mann A, He J, Daon E, Wirtz K, Dalton A, et al. Implementation of an early extubation protocol in cardiac surgical patients decreased ventilator time but not intensive care unit or hospital length of stay. J Cardiothorac Vasc Anesth. 2018;32:739–44. 29229252 10.1053/j.jvca.2017.11.007

[32] Edgerton JR, Herbert MA, Prince SL, Horswell JL, Michelson L, Magee MJ, et al. Reduced atrial fibrillation in patients immediately extubated after off-pump coronary artery bypass grafting. Ann Thorac Surg. 2006;81:2121–7. 16731140 10.1016/j.athoracsur.2006.01.015

[33] Camp SL, Stamou SC, Stiegel RM, Reames MK, Skipper ER, Madjarov J, et al. Quality improvement program increases early tracheal extubation rate and decreases pulmonary complications and resource utilization after cardiac surgery. J Card Surg. 2009;24:414–23. 19583609 10.1111/j.1540-8191.2008.00783.x

[34] Hawkes CA, Dhileepan S, Foxcroft DR. Early extubation for adult cardiac surgical patients. Cochrane Database Syst Rev. 2003;4:CD003587. 10.1002/14651858.CD003587 14583985

[35] Wong WT, Lai VKW, Chee YE, Lee A. Fast-track cardiac care for adult cardiac surgical patients. Cochrane Database Syst Rev. 2016;9:CD003587. 27616189 10.1002/14651858.CD003587.pub3 PMC6457798

[36] Batchelor TJP, Rasburn NJ, Abdelnour-Berchtold E, Brunelli A, Cerfolio RJ, Gonzalez M, et al. Guidelines for enhanced recovery after lung surgery: recommendations of the Enhanced Recovery After Surgery (ERAS®) Society and the European Society of Thoracic Surgeons (ESTS). Eur J Cardiothorac Surg. 2019;55:91–115. 30304509 10.1093/ejcts/ezy301

[37] Li M, Zhang J, Gan TJ, Qin G, Wang L, Zhu M, et al. Enhanced recovery after surgery pathway for patients undergoing cardiac surgery: a randomized clinical trial. Eur J Cardio-Thorac Surg. 2018;54:491–7. 10.1093/ejcts/ezy100 29514224

[38] Subramaniam K, DeAndrade DS, Mandell DR, Althouse AD, Manmohan R, Esper SA, et al. Predictors of operating room extubation in adult cardiac surgery. J Thorac Cardiovasc Surg. 2017;154:1656-1665.e2. 28711332 10.1016/j.jtcvs.2017.05.107

[39] e Silva RAG, Borgomoni GB, Maia A da S, do Vale Juniora CF, Pereira E da S, Silvestre LGI, et al. Extubation in the operating room after coronary artery bypass graft surgery reduces hospital stay. J Cardiothorac Vasc Anesth. 2023;37:1938–45. 10.1053/j.jvca.2023.06.020 37453808

[40] Badhwar V, Esper S, Brooks M, Mulukutla S, Hardison R, Mallios D, et al. Extubating in the operating room after adult cardiac surgery safely improves outcomes and lowers costs. J Thorac Cardiovasc Surg. 2014;148:3101-9.e1. 10.1016/j.jtcvs.2014.07.037. 25173117 10.1016/j.jtcvs.2014.07.037

