# Supplementary material for: Extubation in the operating room after elective on-pump CABG surgery: impact on patient outcome and clinical practice during the COVID-19 pandemic
Source: Indian J Thorac Cardiovasc Surg. 2025 Mar 24;41(7):863–73. doi: 10.1007/s12055-025-01908-9 (PMC12170970; doi:10.1007/s12055-025-01908-9)
Supplement: Supplementary file 1 — Supplementary file1 (DOCX 405 KB) [file 12055_2025_1908_MOESM1_ESM.docx]

^^

^(a)^ due to incomplete documentation, the postoperative left ventricular ejection fraction could not be determined for 4 out of 50 patients in the ITN group, and for 14 out of 47 patients in the EXT group;

^(b)^ due to incomplete documentation, the length of aortic crossclamping could not be included for 3 out of 50 patients in the ITN group, and for 3 out of 47 patients in the EXT group;

^(c)^ due to incomplete documentation, the EURO II score could be calculated for 47 out of 50 patients in the ITN group, and for 44 out of 47 patients in the EXT group.
